# Investigating racial bias within Australian rules football commentary

**DOI:** 10.1371/journal.pone.0272005

**Published:** 2022-07-25

**Authors:** Scott A. MacLeod, Philip W. S. Newall

**Affiliations:** 1 Central Queensland University, Rockhampton, Queensland, Australia; 2 School of Psychological Science, University of Bristol, Bristol, United Kingdom; Newcastle University, UNITED KINGDOM

## Abstract

International research has shown that live sports commentary exhibits racial bias. Specifically, non-White players are more likely to be praised in terms of their physicality, while White players are more likely to be praised in terms of their intellect and character. The current study, which utilised a quantitative content analysis design, examined whether the speech of AFL commentators exhibited racial bias. The study randomly selected 50 men’s AFL game quarters from the 2019 AFL season and analysed 1368 applicable statements directed at 382 unique players. Based on prior research, a coding instrument was developed that incorporated three main categories (physical, cognitive, and character attributes), and six subcategories (physical ability, appearance, cognitive ability, intelligence, general character, and hard work). In contrast to the international literature, findings revealed that there were no significant between-race differences for each main attribute category. However, non-White players received a higher proportion of statements related to their physical ability, and a lower proportion of statements related to their appearance compared to White players. Non-White players also received a higher proportion of negative statements related to their cognitive ability compared to White players. There was no evidence found to suggest that players of any race were discussed in terms of their physical ability being innate, natural, or instinctual. Given the strong, but also dated, evidence showing racial bias within both American and European sports commentary, the current study provides only weak evidence for the existence of racial bias within contemporary AFL live commentary.

## Introduction

Sport plays an important role in Australian culture and national identity [1]. Australian Rules Football, commonly referred to as Aussie Rules or AFL (Australian Football League), is one of Australia’s most-watched sports, with Thursday and Friday night games averaging approximately one million viewers across free-to-air and subscription television [2]. These broadcasts feature live commentary, and the commentator’s role is to entertain the viewer by weaving the on-screen action into a broad and subjective narrative. International research suggests that the speech of sports commentators exhibits racial bias [3]. Specifically, non-White players are more likely to be praised in terms of their physicality, while White players are more likely to be praised in terms of their intellect and character [3, 4]. However, the existence of racial bias within Australian sports commentary, and more specifically within AFL commentary, has yet to be systematically examined.

Racial bias can exist at both the explicit and implicit levels [5]. Explicit racial bias refers to an individual’s consciously held attitudes and beliefs regarding members of a particular racial group. In contrast, implicit racial bias refers to the unconscious and/or automatic mental associations an individual makes between members of a particular racial group and one or more attributes [6]. Live sports commentary provides an excellent real-world environment to examine racial bias, and sports commentators provide a crucial source of biased messages that warrant scholarly study [7]. Commentators are often caught up in the excitement of the game as the fast-paced on-field action occurs. In this often emotionally charged environment, commentators inform and entertain the viewers without having time to select their words carefully. This environment can elicit statements from the commentator that reflect their unconscious beliefs, attitudes, and values [8].

Racial bias, in the context of televised sports commentary, has previously been found in the commentary of several sports, including American football [3, 4, 8], basketball [4, 7], and European soccer [9–11]. Generally, these studies have revealed that non-White athletes are more likely to receive positive comments related to their physical ability, while White athletes are more likely to receive positive comments related to their cognitive ability and character [3]. Such commentary reinforces essentialist thinking and racial stereotypes, namely, that the Black athlete is characterised as being naturally gifted and possessing innate talent, while the success of the White athlete is often attributed to their intelligence and/or hard work [7]. It is vitally important to examine the prevalence of racial bias within AFL commentary for several reasons. Firstly, the speech of commentators is very likely to affect the attitudes of the fans consuming the content. Secondly, the current ingrained habits of AFL commentators are likely to influence future generations of commentators, who seek to emulate their models. And most importantly, these attitudes can extend beyond the realm of sport into the social realm [7].

### Australian rules football

Originally known as Victorian Rules Football, AFL was first played by British colonists in the late 1850s in Melbourne, Victoria. However, there is continued debate among historians as to whether the sport was originally inspired by the Indigenous Australian game of Marngrook [12]. The history of AFL is deeply entwined with Australia’s colonial past. The involvement of Indigenous Australians in sports has been marked by exclusion, discrimination, and gross inequality [13]. It has taken immense courage for many Indigenous Australian athletes to play AFL, overcoming discrimination and prejudice to excel at their chosen sport [14]. Within Australia, the AFL was the first sporting institution to begin the fight against overt racism within team sports, with the introduction of *Rule 30*: *A Rule to Combat Racial and Religious Vilification* in 1995 [14]. This occurred after several well-publicised instances of racial abuse, commonly called racial taunting or sledging, were directed toward Indigenous players by spectators and players from the opposing team [13]. Since this time, there have been several highly publicised instances of overt racism, involving both players and spectators. However, the AFL enforces a strict zero-tolerance policy against racism and vilification of any form [15] and has worked to promote an atmosphere of inclusion within the sport.

Over the past two decades, the AFL has done much to promote Indigenous involvement in the sport through various community programs, academies, and tournaments [14, 16]. More recently, the AFL has also worked to increase engagement and participation rates among Australia’s diverse communities and to promote the sport’s multicultural image. As of 2019, the combined AFL team lists contained 839 players, playing at the elite level of the sport. Of these players, 10% were of Indigenous heritage [17] and a further 15% were of multicultural heritage [18]. However, given the context of the current study, it should be noted that the majority of players of multicultural heritage are of White European heritage [19].

### Racial bias within the Australian sporting context

The research literature clearly outlines the overt racism still experienced daily by Indigenous Australians [20], and the structural disadvantages faced across multiple domains, including health [21], employment, education, and socioeconomic status [22]. The disadvantage and marginalisation faced by Indigenous Australians are also apparent within the institution of the AFL [23]. For example, Aboriginal and Torres Strait Islanders represent 3.3% of the Australian general population [24] and make up approximately 10% of professional AFL players in the league [17]. While Indigenous players are over-represented on the AFL playing field and have been for many years, as of 2018, only three (1.7%) of the 180 AFL coaches were Indigenous Australian [25]. Critical race theorists cite pervasive racial stereotyping as a major factor contributing to the underrepresentation of Indigenous Australians in coaching positions [26], and within positions of leadership and power within the AFL [23].

Only a small number of studies have systematically examined racial bias within the context of AFL, revealing a potential bias against Indigenous players regarding playing time, playing position [13], and the AFL drafting process [27]. Hallinan et al. [13] found that, compared to White AFL players, Indigenous players were on average less likely to be selected to play and were overrepresented in non-central field positions (i.e., playing on the wings or flanks of the game). Non-central field positions, as described by Hallinan et al. [13], often require the stereotypical talents of speed and agility and are viewed as less important positions requiring less responsibility. Similarly, Mitchell et al. [27] found that up-and-coming Indigenous AFL players were discriminated against by being selected for recruitment purposes with lower-order draft picks compared to non-Indigenous players. This is despite the fact that Indigenous AFL players consistently outperformed their matched (i.e., same experience, same draft number, etc.) non-Indigenous peers [27]. In contrast to these findings, Lenten [28] analysed the number of Brownlow Medal votes given to Indigenous and non-Indigenous AFL players between 1998 and 2010. The Brownlow Medal is considered the most prestigious individual award in the AFL, and Lenten [28] sought to examine the possibility of racial bias in the distribution of umpire votes. Surprisingly, results indicated that Indigenous players, on average, received 6.6% more umpire votes compared to their non-Indigenous counterparts [28]. The author of this study noted the novelty of these positive findings in comparison to the general pattern of Indigenous racial discrimination found within both studies examining sport and wider social outcomes [28].

The majority of research carried out in Australia examining racial bias in sports has focused on qualitative analysis from a critical perspective. This literature has described the lived experience of Indigenous athletes and the effects of overt racism on Indigenous Australian team sport players [29]. Furthermore, it has highlighted pervasive essentialist narratives that focus on the Indigenous athlete’s speed or innate talent [30]. These racialized stereotypes, regarding the Indigenous Australian’s predisposed sporting prowess, have been demonstrated within the language of athletes and coaches [31], the Australian media [30], and the language that Indigenous athletes use to describe themselves [23, 32]. Given the pervasive nature of such discourse, one might expect it to be also evident in the speech of AFL match commentary. However, the systematic analysis of live AFL commentary has yet to be carried out within Australia.

### Racial bias within American sports commentary

The majority of research to systematically examine racial bias in televised sports commentary has been undertaken in the United States. One of the earliest studies examined live commentary on American football [33]. That study found that commentary showed more sympathy towards White players and tended to build the positive reputation of White players while doing the inverse toward Black players. The author’s theorized that while the verbalization of the commentator’s beliefs was likely unconscious, the results reflected the White commentator’s prejudicial belief in the superiority of White players and the inferiority of Black players [33].

Rada [8] sought to apply Rainville and McCormick’s [33] techniques to data collected from the 1992 American football season. Rada [8] analysed 586 positive and negative commentator statements, and the pattern of results mirrored that of the earlier study, with all 12 comments that related to sympathy being directed toward White players. Similarly, White players received more comments, and more positive comments, related to their cognitive attributes compared to African American players [8]. While there were no significant between-race differences regarding positive comments relating to a player’s character, all 11 negative comments relating to character were directed toward African American players [8]. In contrast to previous findings, African American players received more play-related praise and play-related criticism from commentators. Furthermore, African American players also received more comments, and more positive comments, related to their physical attributes compared to White players. Rada [8] concluded that this pattern of results in commentary portrayed the African American player as being merely an athlete, praised for physical ability, while the White player was the “thinking man” praised for cognitive ability.

This pattern of results continued to emerge within intercollegiate sports [4], basketball [7], and American football [3]. Rada and Wulfemeyer [4] examined racial bias within the televised commentary on intercollegiate sports. The sample consisted of 486 coded comments made during the 1998 football season, and a championship basketball tournament held in 1999. Findings revealed that African American players were more likely to receive a negative comment (Φ = .15), a comment relating to a physical attribute (Φ = .32), and a negative comment relating to on-field intellect (Φ = .36) compared to White players. In contrast, White players were significantly more likely to receive a positive comment relating to their character (Φ = .22), and on-field intellect (Φ = .12), compared to African American players [4]. Similarly, Eastman and Billings [7] examined 66, male and female, televised college basketball games during the 1999 season. The researchers concluded that racial stereotypes pertaining to the Black athlete as being naturally athletic, and the White athlete as needing to work hard to keep up, were consistently reinforced by commentators. This further reinforces the notion that White athletes possess more cognitive ability and leadership qualities, while Black athletes are lacking in those characteristics [7]. More recently, Merullo et al. [3] confirmed these findings in a large-scale study examining 267,778 comments from 1,455 American football games spanning 59 years. Their analysis showed that non-White players were far more likely to receive positive comments related to their physical ability. In contrast, White players tended to receive positive comments related to their intelligence or personality [3].

### Racial bias within European sports commentary

A similar pattern of results has also been found within the live commentary on European soccer. Examining matches from the 1995–1996 English domestic league, McCarthy and Jones [9] found that Black football players were depicted positively across the three categories of performance, physical characteristics, and psychological characteristics. However, Black players received more positive depictions concerning their physical characteristics, while White players received more positive depictions concerning their psychological characteristics [9]. McCarthy et al. [10] then analysed a larger sample of 100 hours of soccer footage from the 1997–1998 season and found similar results. While Black players still received more positive depictions concerning their physical characteristics the between-race difference regarding psychological characteristics was no longer evident [10].

In a more recent study, conducted by RunRepeat and The Professional Footballers’ Association, McLoughlin [11] examined and coded 2073 commentator statements, from 80 soccer matches, across four of the top leagues in Europe. The ratio adjusted data indicated that when commentators talked about intelligence, players with a light skin tone received 63% of the positive comments, and players with a dark skin tone received 63% of the negative comments. Furthermore, in relation to physical attributes, when a commentator mentioned power, they were 6.59 times more likely to be referring to a player with a dark skin tone. Similarly, when a commentator mentioned speed, they were 3.38 times more likely to be referring to a player with a dark skin tone. Finally, when discussing a player’s work ethic, 60% of the praise was directed toward players of a lighter skin tone [11].

In light of this international research, the current study’s research aim was to examine whether the speech of AFL commentators contains racial bias. To address this, the current study seeks to answer four main research questions:

1. Does the overall frequency of positive and negative AFL commentator statements differ by race?
2. Does the frequency of AFL commentator statements that relate to physical, cognitive, or character attributes differ by race?
3. Does the frequency of positive and negative AFL commentator statements that relate to physical, cognitive, and character attributes differ by race?
4. Does the frequency of AFL commentator statements that refer to players in terms of their innate, natural, or instinctual ability differ by race?

## Materials and method

The study utilised quantitative content analysis, which is a common research methodology based on the systematic coding and quantification of written, visual, or oral content [34]. This methodology was used to examine the speech of AFL commentators during 50 men’s AFL game quarters from the 2019 season. AFL games are structured into four quarters, and each quarter runs for 20 minutes plus time added on for stoppages. Given the frequent stoppages, the average game quarter lasts approximately 30 minutes [35]. Only in-game commentary that occurred between the quarter-time buzzers was coded and analysed, as previous research indicated that a significantly larger proportion of commentary occurs during in-game coverage, compared to pre-game, halftime, or post-game coverage [8].

### Sampling method

The current study examined a random sample of 50 televised AFL game quarters from the 2019 season, which were broadcast on either the Fox Footy or Channel 7 networks. Recordings of the live coverage were accessed via a paid subscription to the official AFL live pass [36]. The sampling method utilised was similar to that employed by previous researchers examining racial bias within sports commentary [8]. This method involves sampling composite games. A composite game consists of four quarters from four different games, potentially involving eight different teams, approximately 176 players, and four commentary teams. This method allows for the inclusion of a wider variety of players, teams, and commentary teams, within a sample of a given size, allowing for more diversity of players and statements [11].

The sample was drawn from the 23 rounds of the normal AFL season. Each game was allocated a number from 1–198 corresponding to the order in which they appeared on the official AFL fixture sheet [37]. Using a random number generator, fifty numbers were generated. For the game corresponding to the first number generated, the first quarter of the game was analysed. For the second number generated, the second quarter was analysed. For the third number generated, the third quarter was analysed. For the fourth number generated, the fourth quarter was analysed. For the fifth number generated, the first quarter was analysed, and so on until the fiftieth number.

### Coding instrument

The coding instrument was developed around the categorical variables utilised in previous research. The majority of studies incorporated broad categories which generally related to a player’s physical [4, 8–10], cognitive [8–10], or character attributes [4, 8]. A number of researchers utilised more refined categories generally related to a player’s work ethic [8, 11], intelligence [4, 11], and appearance [11]. The vast majority of studies also incorporated a positive and negative valance for each category, while the current study also followed this protocol, it is acknowledged that this is value-driven and represents a potential source of bias. To allow for comparison to the majority of previous research, and more in-depth data analysis, the coding instrument was designed to incorporate both main categories (physical, cognitive, and character attributes), and subcategories (physical ability, appearance, cognitive ability, intelligence, general character, and hard work).

The first main category is related to statements made regarding a player’s *physical attributes*. The category of physical attributes incorporated the subcategories of *physical ability* and *appearance*. Physical ability included statements regarding a player’s athleticism, speed, power, stamina, agility, etc. (e.g., “blistering pace by [player] down the wing”), while the subcategory of appearance included statements regarding a player’s size or height (e.g., “[player], he’s the big fella in the middle of the ground”). Given that size and height are important aspects of various on-field roles within AFL, it is important to note that statements simply describing the player’s dimensions, “[player] is 190 centimetres tall”, were not coded. However, if the description included more information, “at 190 centimetres [player] is a big fella”, then it was coded [4].

The second main category is related to statements made regarding, or the attribution of an action to, a player’s *cognitive attributes*. The category of cognitive attributes incorporated the subcategories of *cognitive ability* and *intelligence*. Cognitive ability included statements related to a player’s general cognitive abilities, including awareness, imagination, vision, creativity, play reading, etc. (e.g., “that showed great imagination”), while the subcategory of intelligence included statements directly related to a player’s intelligence and decision making (e.g., “I like him as a player, he’s got good smarts about him”).

The third, and final, main attribute category of *character attributes* is related to statements made regarding, or the attribution of an action to, a player’s character or personality. This category was further broken down into the subcategories of *general character* and *hard work*. General character included statements that related to aspects of a player’s character or personality, including discipline, courage, honesty, etc. (e.g., “he’s so unselfish”), while the subcategory of hard work related specifically to the player’s work ethic and effort (e.g., “you can’t question his work rate”). The full coding instrument (S1 File) is available in the supporting information section of this article. Table 1 shows negative statement examples for each of the three main categories, and six subcategories, of the coding instrument.

**Table 1:** Negative statement examples for each main attribute category and subcategory.

Attribute category & subcategories	Example statement
**Physical attributes**	
Physical ability	“He’s too slow to catch him”.
Appearance	“He’s probably a bit undersized”.
**Cognitive attributes**	
Cognitive ability	“Took his eyes off it at the key moment”.
Intelligence	“He just wasn’t thinking”.
**Character attributes**	
General character	“That was just undisciplined”.
Hard work	“You feel he never really did enough”.

Given the fast-paced nature of AFL commentary, all game quarters were viewed twice. To ensure the reliability of data coding, as recommended by Holsti [38], five games (10%) were randomly selected and dual-coded. To calculate intercoder reliability the Holsti [38] formula was used, and intercoder reliability was calculated at 94.3%. Research indicates that intercoder agreement of above 90% is always acceptable [39].

In addition to the main attribute categories, and attribute subcategories, several other variables were recorded, namely, the player’s team, field position (defence, midfield, forward), and race (White, non-White). Additionally, the network the game was aired on (Fox Footy, Channel 7), and the quarter of the game (1–4), was also recorded.

### Race

To operationalise the variable race, the current study incorporated two methods utilised in previous research. These methods included coding each player’s race from the visual information presented on-screen during the game [4, 9] and the use of official player photographs [3]. Firstly, the race of each unique player contained in the original sample (*n* = 392) was coded by the lead researcher, as either White or non-White, from the visual information presented on-screen during each game. Secondly, to control for any one person’s subjectivities, two additional coders (a White female, and a non-White male) independently coded the race of each unique player from official player photographs contained on their respective teams’ AFL website. In a small number of cases (< 5), an official photograph was not available, in which case a photograph obtained via a Google image search was used [3]. Each coder viewed the photos independently and was given the player’s name and asked to state whether the player was either White or non-White. This process yielded an inter-rater agreement of 91.3% between the three coders. The final stage of race coding involved all three coders jointly re-examining both official player photos, and in-game photos, of the 34 players (8.7%) for which there was an initial disagreement on race between coders. After this final stage, there were ten players for which agreement on race was not reached. Therefore, those ten players were removed from the sample to achieve 100% inter-rater agreement for the race of each unique player contained in the final sample. However, two points should be noted. Firstly, the authors acknowledge that operationalising race as a White/non-White binary exhibits its own kind of prejudice in attempting to classify a range of skin colours and racial identities as if they were alike. While the terms White/non-White are undoubtedly inadequate, this is beyond the scope of the current study given that the focus was to examine perceptions of race from an outside observer (i.e., commentator). Secondly, the possibility of misidentification of race always exists, and it is beyond the scope of this study to enter into a discussion on the biological, anthropological, and sociocultural ramifications of race [4].

### Sample

After transcribing the relevant comments from 50 randomly selected AFL game quarters, this yielded a total of 1400 applicable commentator statements, which were directed at 392 unique players. An additional two commentator statements were not included in the data collection process due to it not being possible to determine the particular player the commentator was referring to. As previously discussed, ten players were removed from the sample, which included the removal of 32 statements, due to coder disagreement on the race of the player.

Several assumptions were tested in accordance with Field’s [40] recommendations on chi-square analysis. Data was collected via random sampling and all categories were mutually exclusive within each analysis. An examination of expected frequencies showed that in the majority of 2x2 analyses no expected count was < 5. However, analysis of the proportion of positive and negative statements, by race, within each attribute subcategory, showed that three cells had expected frequencies < 5. In these instances, a two-sided Fisher’s exact test was performed, and only the *p*-value is reported. In the larger analyses, all expected counts were > 1 and no more than 20% of expected counts were < 5. The assumption of independence was met as each statement contributed to one cell only within each analysis. While the power of each separate analysis varied, a post hoc power analysis of two independent proportions was conducted using G*Power 3.1 [41] for analysis one. The study achieved adequate power of .91 in detecting a small change in effect size of .10, at an alpha of .05.

## Results

### Descriptive statistics

The final sample consisted of 1368 coded commentator statements, 1172 (85.7%) directed at players coded as White, and 196 (14.3%) directed at players coded as non-White. The sample contained 382 unique players, 340 (89%) were coded as White and 42 (11%) were coded as non-White. While official numbers are not available, these figures are representative of the total AFL player base when race is operationalised as a White/non-White dyad. The random sample of 50 televised game quarters contained 33 (66%) quarters that were broadcast on the Fox Footy channel and 17 (34%) that were broadcast on Channel 7. Overall, 1152 (84.2%) statements were coded as positive and 216 (15.8%) were coded as negative. These figures mirror previous findings showing that sports commentators are more likely to provide positive commentary [4]. Finally, players whose field position was in defence received 323 (23.6%) commentator statements, those in midfield 499 (36.5%), and those playing forward 546 (39.9%). The full dataset (S2 File) is available in the supporting information section of this article.

### Analysis 1

The first analysis examined whether the overall proportion of positive and negative AFL commentator statements differed by race. Overall, 84.6% (*n* = 991) of the statements received by White players were positive and 15.4% (*n* = 181) were negative. Similarly, 82.1% (*n* = 161) of the statements received by non-White players were positive and 17.9% (*n* = 35) were negative. A Pearson’s chi-square test of independence showed that there was no significant association between a player’s race and the overall proportion of positive and negative statements received, χ ^2^(1) = 0.74, *p* = .391.

### Analysis 2

The second set of analyses examined whether the proportion of AFL commentator statements received for each main attribute category and subcategory differed by race. These results are displayed in Table 2.

**Table 2:** Proportion of statements received by race for each main attribute category and subcategory.

	Total		White		Non-White	
	*n*	%	*n*	%	*n*	%
Physical attributes	596	43.6	502	42.8	94	48.0
Physical ability	410	30.0	330	28.2	80	40.8*
Appearance	186	13.6	172	14.7	14	7.1*
Cognitive attributes	363	26.5	311	26.5	52	26.5
Cognitive ability	160	11.7	140	11.9	20	10.2
Intelligence	203	14.8	171	14.6	32	16.3
Character attributes	409	29.9	359	30.6	50	25.5
General character	292	21.3	258	22.0	34	17.3
Hard work	117	8.6	101	8.6	16	8.2
Total	1368		1172		196	

*Note*. Percentages do not equal totals due to rounding.

*p < .05 (unadjusted).

A Pearson’s chi-square test of independence showed that there was no significant association between a player’s race and the proportion of statements received for each main attribute category, χ^2^(2) = 2.49, *p* = .289. In contrast, a Pearson’s chi-square test of independence showed that there was a significant association between a player’s race and the proportion of statements received for each attribute subcategory, χ^2^(5) = 18.52, *p* = .002, Cramer’s V = .12. A post hoc analysis of standardised residuals showed that non-White players received a significantly higher proportion of statements related to their physical ability (*z* = 2.77), and a significantly lower proportion of statements related to their appearance (*z* = -2.45), compared to White players.

### Analysis 3

The third set of analyses examined whether the proportion of positive and negative commentator statements received, both for and within each attribute category and subcategory, differed by race. The proportion of positive and negative statements, by race, for each main attribute category and subcategory are displayed in Table 3.

**Table 3:** Proportion of positive and negative statements by race for each main attribute category and subcategory.

	Total				White				Non-White			
	Positive		Negative		Positive		Negative		Positive		Negative	
	*n*	%	*n*	%	*n*	%	*n*	%	*n*	%	*n*	%
Physical attributes	537	46.6	59	27.3	452	45.6	50	27.6	85	52.8	9	25.7
Physical ability	384	33.3	26	12.0	308	31.1	22	12.2	76	47.2*	4	11.4
Appearance	153	13.3	33	15.3	144	14.5	28	15.5	9	5.6*	5	14.3
Cognitive attributes	265	23.0	98	45.4	231	23.3	80	44.2	34	21.1	18	51.4
Cognitive ability	122	10.6	38	17.6	111	11.2	29	16.0	11	6.8	9	25.7
Intelligence	143	12.4	60	27.8	120	12.1	51	28.2	23	14.3	9	25.7
Character attributes	350	30.4	59	27.3	308	31.1	51	28.2	42	26.1	8	22.9
General character	243	21.1	49	22.7	215	21.7	43	23.8	28	17.4	6	17.1
Hard work	107	9.3	10	4.6	93	9.4	8	4.4	14	8.7	2	5.7
Total	1152		216		991		181		161		35	

*Note*. Percentages do not equal totals due to rounding.

*p < .05 (unadjusted).

A Pearson’s chi-square test of independence showed that there was no significant association between a player’s race and the proportion of positive statements (χ^2^(2) = 2.96, *p* = .228), or negative statements (χ^2^(2) = 0.68, *p* = .711), received for each main attribute category. In contrast, a Pearson’s chi-square test of independence showed that there was a significant association between a player’s race and the proportion of positive statements received for each attribute subcategory, χ^2^(5) = 23.45, *p* < .001, Cramer’s V = .14. A post hoc analysis of standardised residuals showed that non-White players received a significantly higher proportion of positive statements related to their physical ability (*z* = 3.05), and a significantly lower proportion of positive statements related to their appearance (*z* = -2.68), compared to White players. However, there was no significant association between a player’s race and the proportion of negative statements received for each attribute subcategory, χ^2^(5) = 2.34, *p* = .800.

The next stage of this analysis examined whether the proportion of positive and negative statements received within each main attribute category differed by race, and these results are displayed in Table 4.

**Table 4:** Proportion of positive and negative statements by race within each main attribute category.

	Total		White		Non-White	
	*n*	%	*n*	%	*n*	%
**Physical attributes**						
Positive	537	90.1	452	90.0	85	90.4
Negative	59	9.9	50	10.0	9	9.6
**Cognitive attributes**						
Positive	265	73.0	231	74.3	34	65.4
Negative	98	27.0	80	25.7	18	34.6
**Character attributes**						
Positive	350	85.6	308	85.8	42	84.0
Negative	59	14.4	51	14.2	8	16.0

A Pearson’s chi-square test of independence showed that there was no significant association between a player’s race and the proportion of positive and negative statements received that related to a player’s physical attributes (χ^2^(1) = 0.01, *p* = .909), cognitive attributes (χ^2^(1) = 1.79, *p* = .181), or character attributes (χ^2^(1) = 0.11, *p* = .735).

The final stage of this analysis examined whether the proportion of positive and negative statements received within each attribute subcategory differed by race, and these results are displayed in Table 5.

**Table 5:** Proportion of positive and negative statements by race within each attribute subcategory.

	Total		White		Non-White	
	*n*	%	*n*	%	*n*	%
**Physical ability**						
Positive	384	93.7	308	93.3	76	95.0
Negative	26	6.3	22	6.7	4	5.0
**Appearance**						
Positive	153	82.3	144	83.7	9	64.3
Negative	33	17.7	28	16.3	5	35.7
**Cognitive ability**						
Positive	122	76.3	111	79.3	11	55.0
Negative	38	23.8	29	20.7	9	45.0*
**Intelligence**						
Positive	143	70.4	120	70.2	23	71.9
Negative	60	29.6	51	29.8	9	28.1
**General character**						
Positive	243	83.2	215	83.3	28	82.4
Negative	49	16.8	43	16.7	6	17.6
**Hard work**						
Positive	107	91.5	93	92.1	14	87.5
Negative	10	8.5	8	7.9	2	12.5

*Note*. Percentages do not equal 100 due to rounding.

*p < .05 (unadjusted).

A Pearson’s chi-square test of independence showed that there was no significant association between a player’s race and the proportion of positive and negative statements received that related to a player’s physical ability (χ^2^(1) = 0.30, *p* = .583), appearance (*p* = .078), intelligence (χ^2^(1) = 0.04, *p* = .847), general character (χ^2^(1) = 0.02, *p* = .886), or hard work (*p* = .625). However, non-White players received a significantly higher proportion of negative statements related to their cognitive ability (*p* = .025, Cramer’s V = .19) compared to White players.

### Analysis 4

The fourth set of analyses sought to examine whether the frequency of AFL commentator statements that referred to players in terms of their innate, natural, or instinctual ability differed by race. However, only three statements were recorded and were thus too few to statistically analyse. Two of the statements were directed at White players, “he looks a more natural forward” and “instinctively he hand balls”. One statement was directed at a non-White player, “not really in [Player]’s DNA to be so conservative”.

## Discussion

The current study aimed to examine whether the speech of AFL commentators exhibited racial bias. Regarding the current study’s first research question, “does the overall frequency of positive and negative AFL commentator statements differ by race?”, results indicated that there were no significant between-race differences in the overall proportion of positive and negative AFL commentator statements. Regarding the second research question, “does the frequency of AFL commentator statements that relate to physical, cognitive, or character attributes differ by race?”, no significant between-race differences were found in the proportion of statements received for each main attribute category. However, analysing the data in the form of six subcategories indicated that non-White players received a significantly higher proportion of statements related to their physical ability and a significantly lower proportion of statements related to their appearance. Regarding the third research question, “does the frequency of positive and negative AFL commentator statements that relate to physical, cognitive, and character attributes differ by race?”, there were no significant between-race differences found in the proportion of positive and negative statements received for each main attribute category or within each main attribute category. However, analysing the data as six subcategories indicated that non-White players received a significantly higher proportion of positive statements related to their physical ability, and a significantly lower proportion of positive statements related to their physical appearance, compared to White players. Non-White players also received a higher proportion of negative statements related to their cognitive ability compared to White players. Finally, there were insufficient data to statistically analyse the fourth research question.

### Overall positive and negative statements

The current study found no significant between race differences in the overall proportion of positive and negative statements, with 15% and 18% of statements being negative for White and non-White players, respectively. While previous researchers tended to not report the overall figure, some rudimentary calculations showed that in the European context the percentage of negative statements ranged between 24% and 27% for both White and non-White players [9–11]. In contrast, in the American context, the overall percentage of negative statements ranged between 4% and 5% for White players, and 12% and 17% for non-White players [4, 8]. Therefore, findings suggest that Australian AFL commentators are slightly less negative than their European counterparts, and while they appear to be slightly more negative than their American counterparts, their overall commentary does not exhibit a similar degree of racial bias.

### Physical attributes

The majority of prior research utilised a single variable to represent a player’s physical attributes that incorporated both statements related to physical ability and physical appearance. All of these studies, beyond the original Rainville and McCormick [33] study, found that non-White players received a higher proportion of statements [4], and/or positive statements [4, 8–10], related to their physical attributes. In contrast, the current study found no between-race differences regarding the main category of physical attributes.

One possible explanation for this finding may be unique to the Australian context, namely, that reference to an individual’s size is common in Australian slang and thus in Australian sports commentary. For example, “he’s the big fella out the back”, “look at the little champ go”, big Johnno’s got the ball”, etc. The high frequency of such statements related to appearance may obscure meaningful differences when combined with physical ability under a single construct of physical attributes. Only one prior study [11] examined the category of appearance in isolation. However, only five statements were recorded and were thus too few to be meaningful. This is in stark contrast to the current study which found that 14% (*n* = 186) of recorded statements referred to a player’s appearance, illustrating the AFL commentator’s tendency to refer to a player’s size. Therefore, combining aspects of physical ability and appearance under a single variable, as was the case within much of the prior research, may obscure meaningful differences in the Australian context of sports commentary. These meaningful differences became apparent when examining the data in the form of subcategories.

The finding that non-White players received a significantly higher proportion of commentary related to their physical ability supports the most robust finding within the international literature and certainly points to the possibility of racial bias in the Australian context. Furthermore, relative to other categories, non-White players also received a higher proportion of positive comments for the subcategory of physical ability. However, interestingly, this difference in positive commentary is driven by the commentators’ tendency to deliver positive commentary, combined with their tendency to discuss non-White players in terms of their physical ability. It is not the case that non-White players are discussed more positively, compared to White players, regarding their physical ability. This finding is in contrast to previous research which consistently found that non-White athletes were discussed more positively in terms of their physical attributes and/or physical ability [4, 8–11].

Additionally, while non-White players received a significantly higher proportion of statements related to their physical ability, they also received a significantly lower proportion of statements, and positive statements, related to their physical appearance, compared to White players. There is one possible explanation, that may explain this overall pattern of results, that certainly warrants further investigation. Specifically, bigger players may tend to be referred to in terms of their size and height (i.e., appearance), and a higher proportion of bigger players are White. In contrast, smaller players may tend to be referred to in terms of their speed and agility (i.e., physical ability), and relatively speaking, a higher proportion of smaller players are non-White. For example, the average height of a player in the AFL is approximately 6.2ft (188cm) [42]. However, 76% of the 42 non-White players contained in the current study’s sample are listed as having a height of 6.1ft (186cm) or less. This may also explain why non-White players received a significantly lower proportion of positive commentary in relation to their appearance, as being referred to as small was coded as negative. Thus, a player’s height, as opposed to their race, may better predict commentator statements related to physical ability and appearance within the Australian context. This is discussed further in the limitations and future research section.

### Cognitive attributes

The current study found that, within the subcategory of cognitive ability, non-White players received a significantly higher proportion of negative statements compared to White players. This finding is in-line with the majority of prior research which tended to show that non-White players were discussed more negatively in terms of their intelligence or cognitive attributes [4, 8, 9, 11]. However, the current study’s overall results, regarding cognitive attributes, are decidedly mixed in light of these fairly consistent findings. If racial bias were indeed present, one would also expect to find significant between-race differences within the main category of cognitive attributes and/or within the subcategory of intelligence. However, this was not the case and this singular finding in isolation should be taken with caution, especially given the low frequencies within this subcategory. This point is discussed further within the limitations section. The overall results regarding cognitive attributes, cognitive ability, and intelligence appear to align more closely with McCarthy et al. [10] who found no significant between-race difference concerning psychological descriptors.

### Character attributes

The current study found no significant between-race differences in either the proportion of statements received or in the proportion of positive and negative statements received, for the main category of character attributes. This finding also held true when the data were examined as subcategories (general character and hard work). Between-race differences regarding commentator statements related to character have the least empirical support within the literature, among the variables examined within the current study. While Rada and Wulfemeyer [4] found that White players received a higher proportion of positive comments in relation to character, and both Rada and Wulfemeyer [4] and Rada [8] found that non-White players received a higher proportion of negative comments in relation to character, the frequencies were too low to statistically analyse. And in the European context, a variable representing a player’s character has yet to be examined.

In relation to hard work, Rada [8] found no between-race differences in statements regarding whether players achieved results through hard work or God-given ability. In the European context, McLoughlin [11] reported that light skin tone players received a higher proportion of statements in relation to hard work. However, an examination of McLoughlin’s [11] raw numbers showed that approximately 12% of the statements, in relation to hard work, were negative for both light and dark skin players. Thus, while players with a light skin tone received a slightly higher proportion of commentary in relation to hard work they were not discussed more positively. Therefore, the current study’s finding that there were no between-race differences in relation to statements regarding a player’s character or work ethic appears to align with the lack of significant results found within prior research.

### Innate, natural, or instinctual ability

Only three statements were recorded that made reference to any aspect of a player in terms of being innate, natural, or instinctual. Two of the statements were directed at White players, and one statement was directed at a non-White player. None of these statements directly referred to a player’s physical ability, in terms of being innate or natural. This is in contrast to the considerable amount of sociological research [23, 30–32] that demonstrates the pervasive essentialist stereotype, regarding the Indigenous Australian athlete’s innate sporting prowess, that is evident within various other media contexts. On the whole, it appeared that such language was largely absent from contemporary AFL commentary. Although, it could be the case that some commentators do indeed endorse this essentialist stereotype regarding the Indigenous Australian athlete. However, they may be guarded against using such language due to the professional and social ramifications of holding this view.

### Limitations

The current study examined differences between the commentary contained on both of the networks on which AFL is televised (i.e., Fox Footy and Channel 7). Each AFL game generally involved rotating teams of four or five individual commentators, involving over 40 different commentators, across the two networks [43]. Given the large number of commentators, and the difficulty distinguishing between the commentators’ voices during the fast-paced sections of commentary, it was decided to assess possible differences between broadcasting networks, as opposed to possible differences between individual commentators. However, only analysing the broad category of network did not allow for the assessment of possibly racially biased statements made by individual commentators.

A second limitation, which is also present within the majority of previous research, relates to the operationalization of the variable race. As previously mentioned, attempting to classify a diverse range of racial identities into the binary skin colour categories of White/non-White is reductive and exhibits its own kind of prejudice. Furthermore, while such binary classifications are generally utilised in order to achieve sufficient power for statistical analysis, skin colour does not equate to race.

A further limitation, also highlighted by Merullo et al. [3], regards the small data sets generally used within research into racial bias within sports commentary. The current study utilised an adequate sample size relative to most prior studies and had sufficient power. However, there is the possibility that small between-race effects (.10) exist and were undetected by the current study.

A final limitation that must be noted, which is inherent within exploratory research of this nature, regards the study’s family-wise error rate. Performing in-depth and detailed analysis of data, which involved multiple significance tests, inflates the type I error rate. Thus, the *p*-values presented in the paper are purely nominal and have not undergone any form of correction (i.e., Bonferroni correction) to account for the large number of significance tests performed on the data.

### Future research

Given the exploratory nature of the current study, it is suggested that future research is required in order to replicate these findings. However, there is an evident need for future research to adopt a more sophisticated analysis of race beyond an individual’s appearance and skin colour. Particularly, when research is conducted within multicultural societies, with diverse populations, such as Australia. It is also suggested that future research utilises a matched-pairs design. Firstly, matching White and non-White AFL players, on both field position and height/size, would allow for control of the effects of field position. Secondly, this would allow for analysis of the possible mediating effects of a player’s height/size on the relationship between race and statements related to physical ability, and race and statements related to appearance. Alternatively, player height and/or size could simply be added as covariates within a regression analysis. Finally, it is important for future research to assess the impact of, and interaction between, race and gender within live sports commentary. Thus, the systematic analysis of Women’s AFL commentary warrants future study.

## Conclusion

In summary, the current study systematically examined the speech of AFL live commentary to assess whether live commentary, in the Australian context, exhibited racial bias. The results of this exploratory study found that non-White players were no more likely to be discussed in terms of their physical attributes (i.e., body), nor were they discussed in more positive terms regarding their physical ability, nor were their abilities referred to as being innate or instinctual. Furthermore, there were no between-race differences in commentary related to intelligence, cognitive attributes, character, or work ethic. However, non-White players were more likely to be discussed in terms of their physical ability and were less likely to be discussed in terms of their appearance compared to White players. Non-White players also received more negative commentary in relation to their cognitive ability compared to White players. These results provide only weak evidence to support the existence of racial bias, in the context of contemporary AFL commentary, compared to the previous findings from international research.

## Supporting information

S1 File Coding instrument. (DOCX)

S2 File Dataset. (XLSX)
