# Supplementary material for: Investigating racial bias within Australian rules football commentary
Source: PLoS One. 2022 Jul 25;17(7):e0272005. doi: 10.1371/journal.pone.0272005 (PMC9312403; doi:10.1371/journal.pone.0272005)
Supplement: S1 File — (DOCX) [file pone.0272005.s001.docx]

**S1 Codebook**

The codes assigned to each variable, and each variable category, are displayed in Table S1.

**Table S1. Codes assigned to each variable and variable category.**

| **SPSS name** | **Variable** | **Coding instructions** | **Measurement scale** |
| --- | --- | --- | --- |
| Case | Case number | Number assigned to each entry | Scale |
| GameID | Game identification number | Number assigned to each game | Nominal |
| Quarter | Game quarter | 1 = 1^st^ Quarter | Nominal |
|  |  | 2 = 2^nd^ Quarter |  |
|  |  | 3 = 3^rd^ Quarter |  |
|  |  | 4 = 4^th^ Quarter |  |
| Bcast | Broadcaster | 1 = Fox Footy | Nominal (binary) |
|  |  | 2 = Channel 7 |  |
| Team | Team name | 1 = Adelaide Crows | Nominal |
|  |  | 2 = Brisbane Lions |  |
|  |  | 3 = Carlton |  |
|  |  | 4 = Collingwood |  |
|  |  | 5 = Essendon |  |
|  |  | 6 = Fremantle |  |
|  |  | 7 = Geelong |  |
|  |  | 8 = Gold Coast Suns |  |
|  |  | 9 = GWS Giants |  |
|  |  | 10 = Hawthorn |  |
|  |  | 11 = Melbourne |  |
|  |  | 12 = North Melbourne |  |
|  |  | 13 = Port Adelaide |  |
|  |  | 14 = Richmond |  |
|  |  | 15 = St Kilda |  |
|  |  | 16 = Sydney Swans |  |
|  |  | 17 = West Coast Eagles |  |
|  |  | 18 = Western Bulldogs |  |
| Position | Player position | 1 = Defence | Nominal |
|  |  | 2 = Midfield |  |
|  |  | 3 = Forward |  |
| Race | Player race | 1 = White | Nominal (binary) |
|  |  | 2 = Non-White |  |
| Valence | Statement valence | 1 = Positive | Nominal (binary) |
|  |  | 2 = Negative |  |
| Att | Attribute category | 1 = Physical | Nominal |
|  |  | 2 = Cognitive |  |
|  |  | 3 = Character |  |
| AttSub | Attribute subcategory | 1 = Physical ability | Nominal |
|  |  | 2 = Appearance |  |
|  |  | 3 = Cognitive ability |  |
|  |  | 4 = Intelligence |  |
|  |  | 5 = General character |  |
|  |  | 6 = Hard work |  |

**General Rules**

1. Statements made only within game quarters are recorded. Comments made before or after the quarter-time buzzers are excluded.
2. Statements made about any players that are not playing in the current game are excluded.
3. Statements referring to the whole team or multiple players are excluded. For example, “[team], they’re not going to give up, that’s for sure”, “the back row is tiring”, “the two big men”, “look at the work rate of [player A], [Player B], and [player C]” etc.
4. Statements that are judged to be neutral and that simply describe play are excluded. For example, “[player], running down the wing”, “[player A], passes to [player B]”, “tackled by [player]”, “[player] drops the ball” etc.
5. Statements referring to a player’s skill or skill level are excluded. For example, “excellent pass”, “poor pass by [player]”, “well done by {player}” etc.
6. Statements referring to play-related mistakes and errors are excluded (e.g., “fumble by [player]”). Unless the statement also specifically refers to an attribute (e.g., “fumble by [player], that was a lack of concentration”).
7. Statements simply referring to game statistics are excluded (e.g., “he’s had two disposals and one goal already this quarter”). Unless the statement also specifically refers to an attribute (e.g., “twelve disposals this quarter, he’s working really hard”).
8. Statements referring to the player’s performance, or form are excluded (e.g., “excellent performance by [player] tonight”). Unless the statement also specifically refers to an attribute (e.g., “courageous performance tonight by [player]”).
9. Statements referring to the influence or impact of the player on the game or team are excluded (e.g., “he’s really influenced the game”). Unless the statement specifically refers to an attribute (e.g., “[players] speed has had a real impact on the game”).
10. Only statements referring to, or with inference to, a player’s physical, cognitive, or character attributes are recorded.
11. If the same commentator repeats a statement, or makes a very similar statement, regarding the same specific player attribute (e.g., all regarding speed, or all regarding confidence), within the same sentence of speech, only one is recorded. For example:

Commentator 1 – “[player] is fast, he’s fast”.

Commentator 1 – “That was well thought through, a clever little kick”.

However, if a second commentator makes a similar statement, regarding the same attribute, both are recorded. For example:

Commentator 1 – “[player] is flying down the wing”.

Commentator 2 – “he’s just so fast”.

Or if the same commentator repeats a statement, or makes a similar statement, in a later sentence of speech both are recorded.

1. If a commentator makes a statement containing references to more than one attribute (or attribute subcategory), both are recorded under their applicable attribute. For example:

Commentator 1 – “he’s a big, strong player”.

Commentator 1 – “he’s such a smart player and a hard worker too”.

1. To control for multiple similar statements, a maximum of three statements only will be recorded, if/when all the commentator statements relate to the same player, the same specific attribute (e.g., all regarding speed, or all regarding confidence), and the same incident/discussion.

**Kicking**

Statements referring to the apparent strength or power of any kick are viewed as a player skill and are not recorded. Additionally, statements that refer to a quick or fast kick are viewed as just describing the short amount of time the player held onto the ball and are not recorded. The only statements that are recorded in relation to kicking are those that also specifically refer to an attribute (e.g., “that was a brave kick”, “very clever kick” etc).

**Leaping or jumping**

Any jump or leap described as big, high, huge, [player] flies, etc. is counted as describing the leaping ability of the player. Noting that on occasion the term “flies” can simply refer to the act of jumping while running and is not necessarily referring to the height of the jump.

**Tackling or attacking**

Any tackle or attack on the ball described as big, huge, strong, powerful, etc. is counted as describing the tackling players’ strength.

**Passing**

Statements that refer to a quick pass, quick release, quick knock-on, etc. are seen as describing the short amount of time the player held onto the ball and are not recorded. The only statements that are recorded in relation to passing are those that also specifically refer to an attribute (e.g., “that was a brave pass”, “very clever knock-on” etc.). Furthermore, statements that refer specifically to fast or quick hands are counted as speed of body.

**Running**

Any run, or acceleration, described in a non-neutral manner that infers speed (e.g., [player] got away from him with ease”, “bursting away” etc.) are counted as describing the player’s speed.

**Marking**

Any mark described as “strong” is counted as describing the marking player’s strength.

**Play reading**

Within the context of the majority of team sports, “play reading” could be conceptualised under the subcategory of intelligence. Given that the reading of play generally requires detailed knowledge of the sport, tactics, and the accurate prediction of one’s teammates performing certain actions. While this form of play reading undoubtedly occurs frequently within AFL, the ariel nature of the sport makes it impossible to delineate between commentator statements that refer to the reading of the play and the reading of ball flight. As the phrases “read it well” and “read the play” are used frequently in both instances. Therefore, commentator statements referring to play reading are conceptualised under the cognitive attribute subcategory of cognitive ability as opposed to the subcategory of intelligence.

**Attribute categories and subcategories**

**Physical attributes**

**Description**

Any positive or negative statement that refers to a player’s physical attributes, physical ability, or bodily appearance. This category is further broken down into the two subcategories of physical ability and appearance.

**Physical ability**

**Inclusion criteria**

Any positive or negative statement that refers to a player’s athleticism, acceleration, speed, strength, power, agility, jumping/leaping ability, and stamina/fatigue.

**Exclusion criteria**

Any positive or negative statement referring to a player’s physical appearance.

**Positive examples**

“huge leap by [player]” “[player] flies” (as in jumping very high)

“[player] with a burst, cruising past” “[player] with a surge of speed”

“[player] attacking strongly” “he’s a strong-bodied player”

“[player] runs past with speed” “big tackle”

**Negative examples**

“[player] was too slow to catch him” “[player] was out muscled”

“he drops it, fatigue again” “you’re right about [player] tiring”

“he’s done for the day, he’s maxed out”

**Appearance**

**Inclusion criteria**

Any positive or negative statement that refers to a player’s physical appearance (of body, or body parts), including height, weight, or size.

**Exclusion criteria**

Statements that simply describe a player’s dimensions (e.g., “[player] is six foot three and 120 kgs”) are not recorded. However, “[player] is massive at six foot three and 120kg’s” would be recorded.

**Positive examples**

“he’s the big fella in the middle of the ground” “he has the height advantage”

“[player] uses his enormous frame” “he was just too big”

**Negative examples**

“what can the little fella do here” “he’s the smallest guy down there”

**Cognitive attributes**

**Description**

Any positive or negative statement that refers to, or attributes an action to, a player’s cognitive attributes. This category is further broken down into the two subcategories of cognitive ability and intelligence.

**Cognitive ability**

**Inclusion criteria**

Any positive or negative statement that refers to, or attributes an action to, a player’s cognitive ability (other than intelligence). For example, reference to player’s concentration, decisiveness/hesitation, awareness, imagination, vision, creativity, etc. The ability to read play and ball flight is also included under this criteria.

**Exclusion criteria**

Any statement related to an individual’s intelligence, thinking, or decision-making.

**Positive examples**

“that showed great imagination” “goes long, great vision”

“brilliant awareness” “he read it well”

**Negative examples**

“he had a mental lapse” (in concentration) “[player] just read it better than him”

“he hesitated there” “he just took his eye off the ball”

**Intelligence**

**Inclusion criteria**

Any positive or negative statement that refers to, or attributes an action to, a player’s intelligence, thinking, or decision making.

**Exclusion criteria**

Any positive or negative statement that refers to, or attributes an action to, a player’s concentration, decisiveness, awareness, imagination, vision, creativity, etc. Or a reference to the ability to read play and ball flight.

Statements regarding play-related mistakes or errors are excluded (e.g., “he went for the long ball and shanked it”). Unless described or discussed in the context of being the wrong option, choice, or decision (e.g., he went for the long ball, that was the wrong option”).

References to risky or dangerous kicks/plays are excluded (e.g., “risky kick by [player]”). Unless described or discussed in the context of being the wrong option, choice, or decision (e.g., risky kick by [player], bad decision).

**Positive examples**

“I like him as a player, he’s got good smarts” “clever kick”

“that was the right option from [player]” “[player] is still there, he kept his wit’

“all he can do is kick down the line, smart play” “he made the right choice there”

**Negative examples**

“what was he thinking, indescribably bad” “bad option”

“the kick was indescribable….strange decision” “he should have passed it to [player]”

**Character attributes**

**Description**

Any positive or negative statement that refers to, or attributes an action to, a player’s character, personality, or work ethic. This category is further broken down into the two subcategories of general character and hard work.

**General character**

**Inclusion criteria**

Any positive or negative statement that could be considered to relate to a player’s character or personality traits. These include comments related to confidence, honesty, determination, commitment, competitiveness, reliability, composure, courage (gutsy), discipline, etc.

This category also includes phrases that could be considered to denote the character of a person, such as, “a good sportsman”, “equal to the moment”,” rising to the occasion”, “held his nerve” etc.

References to leadership qualities are also considered to denote character. However, statements that simply state that the player is the Captain or leader are excluded.

The word “hard” (e.g., “fights hard”, “goes in hard”) is taken to refer to the determination of a player. Unless the meaning is clearly referring to strength (e.g., “wow, that was a hard tackle”).

**Exclusion criteria**

Any positive or negative statement that refers to a player’s work ethic, work rate, hard work, or effort.

**Positive examples**

‘[player], he never gives up in a contest” “he’s so unselfish”

“[player], nothing was gonna stand in his way” “he showed courage there”

“look at the poise of the champ” “he stood his ground and took the hit”

**Negative examples**

“I think he just lacked the confidence” “didn’t have the confidence to go long”

“he needs to put up more of a fight” “he wasn’t committed to the tackle”

**Hard work**

**Inclusion criteria**

Any positive or negative statement that refers to a player’s work ethic, work rate, hard work, contribution, or effort.

**Exclusion criteria**

The term “effort” judged to be in relation to a player’s “attempt” (usually at scoring) is excluded.

The term “effort” judged to be in relation to a player’s determination, as opposed to hard work, would be included under the subcategory of general character.

The terms “good work” and “good effort” judged to be used in the context of Australian slang to denote doing a good job (i.e., skill), are excluded.

**Positive examples**

“[player], he’s working really hard” “[player], is everywhere”

“you can’t question his work rate” “he’s the engine room of the midfield”

**Negative examples**

“You feel he never really did enough as a player” “he’s just not putting in the effort”

**Innate ability**

Any statement referring to a player as having innate, natural, or instinctual ability will be recorded separately.

**Examples**

“He looks so natural” “that was just instinctual”

“he’s a natural talent” “wow, that’s just innate ability”
